# Rabbit Lines Divergently Selected for Total Body Fat Content: Correlated Responses on Growth Performance and Carcass Traits

**DOI:** 10.3390/ani10101815

**Published:** 2020-10-06

**Authors:** Rozália Kasza, Tamás Donkó, Zsolt Matics, István Nagy, Ádám Csóka, György Kovács, Zsolt Gerencsér, Antonella Dalle Zotte, Marco Cullere, Zsolt Szendrő

**Affiliations:** 1 Faculty of Agricultural and Environmental Sciences, Kaposvár University, Guba S. Str. 40, H-7400 Kaposvár, Hungary; kasza.rozalia@ke.hu (R.K.); matics.zsolt@ke.hu (Z.M.); nagy.istvan@ke.hu (I.N.); csoka.adam@sic.medicopus.hu (A.C.); gerencser.zsolt@ke.hu (Z.G.); szendro.zsolt@ke.hu (Z.S.); 2 Medicopus Nonprofit Ltd., Guba S. Str. 40, H-7400 Kaposvár, Hungary; 3 Analytical Minds Ltd, Árpád Str. 5, H-4933 Beregsurány, Hungary; gyuriofkovacs@gmail.com; 4 Department of Animal Medicine, Production and Health, University of Padova, Agripolis, Viale dell’Universitá 16, 35020 Legnaro, Italy; antonella.dallezotte@unipd.it (A.D.Z.); marco.cullere@unipd.it (M.C.)

**Keywords:** growing rabbit, divergent selection, computed tomography, live performance, carcass fatness

## Abstract

**Simple Summary:**

The aim of this study was to examine the effectiveness of selection for total body fat content and its effect on productive and carcass traits. Growing rabbits were selected for high or low body fat content. The selection was effective, and the difference in fat reserves (perirenal fat and scapular fat content) increased with each generation. After four generations, the feed conversion rate improved, and the ratios of fore and hind parts increased in lean rabbits. Selection for lower total body fat content could be useful for customers who want to buy animals and meat with lower fat content; while selection for higher fat content could be advantageous for rabbit does because they have more fat (energy) deposits which are in positive connection with maternal ability and a longer lifespan.

**Abstract:**

The aim of this experiment was to study the effect of divergently selected rabbits for total body fat content (fat index) on growth performance and carcass traits. The fat index was determined at 10 weeks of age by computed tomography and lasted for four consecutive generations. The rabbits with the lowest fat index belonged to the lean line and those of the highest values belonged to the fat line. At generation four, 60 rabbits/line were housed in wire-mesh cages and fed with commercial pellet ad libitum from weaning (5 w of age) to slaughtering (11 w of age). Growth performance, dressing out percentage and carcass adiposity were measured. The lean line showed a better feed conversion ratio (*p* < 0.001) than the fat line. Furthermore, the carcass of the lean rabbits had the highest proportion of fore (*p* < 0.020) and hind (*p <* 0.006) parts. On the contrary, rabbits of the fat line had the highest carcass adiposity (*p <* 0.001). The divergent selection for total body fat content showed to be effective for both lean and fat lines. Selection for lower total body fat content could be useful for terminal male lines, while the selection for higher total body fat content could be an advantage for rabbit does in providing fat (energy) reserves.

## 1. Introduction

In recent decades, the main trends in rabbit selection programmes were the increase in doe prolificacy in maternal lines, and the maximization of the growth rate in terminal lines. The first purpose was aimed at weaning a higher number of kits, as it represents one of the most important economic components of intensive meat production. The second purpose was aimed at anticipating the slaughter weight [1]. More recently, selection programmes have also included traits such as longevity, the homogeneity of birth weight, residual feed intake, meat yield, and resistance to diseases [2,3,4].

The effect of the divergent selection for fat content has been examined in many animal species. Hetzer and Harvey [5] were pioneers in this sense. They selected two different pig breeds for backfat thickness over 8 and 10 generations and demonstrated in both cases that the selection process was rather effective in both directions. In poultry, Cahaner et al., Zhang et al.; Baéza and Le Bihan-Duval [6,7,8] provided successful examples of divergently selected chicken lines for abdominal fat deposition during 2, 7 and 11 generations, respectively.

In the rabbits farmed for their meat, Martínez-Álvaro et al. [9] divergently selected for intramuscular fat (IMF) content in the Longissimus thoracis et lumborum muscle at 9 weeks, and they obtained satisfactory results after six generations of selection. The rabbits were also divergently selected for thigh muscles volume by in vivo computed tomography (CT) scans, and significant responses were obtained after two generations: together with an increase in thigh muscle volume, the fat volume was also concurrently modified [10] highlighting an inverse meatiness–fatness relationship. In mice, as a model animal, Martinez et al. and Bünger et al. [11,12] examined the long-term effect of the divergent selection for the ratio of gonadal fat pads to body weight, or for body fatness during 20 and 60 generations, respectively.

As known, the body contains four adipose depots: visceral, subcutaneous, intermuscular and intramuscular. Intramuscular fat content influences some sensory traits such as flavour, juiciness and tenderness, which are important components of meat hedonic acceptability [13]. Subcutaneous fat can be an energy stock, but it also plays a major role as an insulator, thus protecting against cold [14]. The main role of visceral fat is energy deposit. In rabbits, the perirenal fat is the main part of visceral fat depots, representing about 50–60% of the total carcass fat content [15]. When animals are underfed, they mobilize their body reserves which are recovered when sufficient food is available. In farmed mammals, malnutrition can also occur during pregnancy and lactation, when animals are unable to consume the amount of feed they need, therefore forcing them to mobilize their fat reserves [16].

In rabbits, a higher or lower body fat content can both be beneficial and detrimental. Low fat content in meat is preferred by consumers [13], so a divergent selection towards a leaner meat is in favour of their request.

If the target is the animal, in particular the rabbit doe, a higher body fat content is sought to limit the negative energy balance during the last phase of pregnancy, and near the lactation peak.

If the target is the growing rabbit, high body fat content is disadvantageous since the incorporation of adipose tissue requires more feed than that needed for the incorporation of lean tissue, leading to a worsening of the feed conversion ratio [10]. In addition, the dissectible fat represents a slaughter waste to be disposed of.

Based on these premises, the present research investigated the effect of a rabbit divergent selection for total body fat content by the means of CT technology on growth performance and carcass traits.

## 2. Materials and Methods

This study was approved by the Ethical Committee of Kaposvár University (Hungary). All animals were handled according to the principles stated in the EC Directive 86/609/EEC regarding the protection of animals used for experimental and other scientific purposes [17].

### 2.1. Animals, Computed Tomography Measurement, Housing and Feeding

The experiment was carried out at the Kaposvár University (Hungary) on the maternal line of the Pannon Ka genetic line [18]. The main selection trait of the breed, since its establishment in 1999, is the number of kits born alive. For the purpose of this study, the divergent selection process was based on the fat index calculated in live rabbits aged 10 weeks, calculated as the ratio of the total body fat volume (cm^3^), the latter estimated by computed tomography (CT), to the body weight (kg). The cross-sectional imaging was performed at the Kaposvár University, Institute of Diagnostic Imaging and Radiation Oncology by a Siemens Somatom Sensation Cardiac type multidetector CT equipment, using the following settings: tube voltage: 120 kV, current: 140 mAs, and the data collection mode was spiral with pitch factor 1 and a field of view of 500 mm. For the CT examination, rounds of three rabbits were fixed with belts in a special plastic container, without using any anaesthetic. The CT measurements consisted of overlapping 2 mm-thick slices covering the whole body. After the acquisition, images were reconstructed with full coverage. Voxels between −200 and −20 Hounsfield Units were considered fat according to Romvári et al. [19] and McEvoy et al. [20]. The fat voxels of the whole body were summarized, then decreased by morphologic tools (erosion) due to the partial volume effects—predominantly on the boundary of the skin and air [21,22]. The cross-sectional digital imaging process was performed according to ISO 9001:2015 quality management system and ISO 14001:2015 environmental management system. The rabbits with the lowest fat index formed the lean selected line, whereas those with the highest fat index formed the fat selected line. The number of CT-scanned rabbits and those selected as reproducers (based on CT results) at each generation, is shown on Table 1. About 33–70% of CT-scanned females and 32–50% CT-scanned males were selected as fat or lean breeding rabbits.

Fat and lean rabbit does were inseminated with the semen from selected bucks of the same line. In each generation, the scapular and perirenal fat were separated and weighed on slaughtered rabbits from lean and fat lines, in order to monitor the dissectible fat change with each generation. The results for growth performance and carcass traits of generation 1 and 3 have been published [23,24].

Rabbits from the lean and fat lines of the 4th generation of divergent selection (60 animals per line) were housed in wire-mesh cages (62.5 cm × 32 cm × 30 cm; 3 rabbits/cage; 16 rabbits/m^2^) and fed with commercial pellet ad libitum from weaning (5 w) to slaughter (11 w). Two commercial diets were offered to the rabbits: one between 5 and 9 weeks of age (15.7% crude protein, 2.4% ether extract, and 9.9 MJ of digestible energy/kg feed) and one during last two weeks of farming (16.3% crude protein, 3.8% ether extract and 10.6 MJ of digestible energy/kg feed). Drinking water was available ad libitum from nipple drinkers. The daily lighting period was 16 hours and the average temperature was 15–18 °C.

From 5 to 11 weeks of age, the individual body weight (BW) of rabbits and the feed intake per cage unit were measured weekly. Thereafter, the daily weight gain (DWG), daily feed intake (FI) and the feed conversion ratio (FCR) were calculated. Mortality did not occur during the growing period.

### 2.2. Slaughter Procedure

At 11 weeks of age, all rabbits were transported to the commercial slaughterhouse located 200 km away from the rabbit farm. Prior to slaughter the rabbits were starved for 6 h (including transport), then they were weighed, and slaughtered after electric stunning. After bleeding out and the removal of the skin and the digestive tract, the hot carcasses (HCW) (with head, kidneys, liver, thoracic cage organs and fat depots) were weighed. Then, after a 24 h cooling at 3–4 °C, the chilled carcasses (CCW) were weighed again and dissected according to recommendation of the WRSA [25]. The weight of the head, liver, kidneys, thoracic cage organs, reference carcass (RCW:CCW minus the set of organs, liver and kidneys), perirenal fat, scapular fat, fore-, mid- and hind parts of the carcass, were recorded. The dressing out percentages (DoP) were calculated for hot, chilled and reference carcasses (HC, CC and RC, respectively). The ratio on the chilled carcass was calculated for RC, head, thoracic cage organs, liver, and kidneys. The ratios on the RC were calculated for the different RC cuts (fore, mid, and hind cut) and fat depots (perirenal fat, scapular fat, and dissectible fat).

### 2.3. Statistical Analysis

The data were analysed by PROC MIXED procedure using SAS 9.4 software package [26], for all carcass traits the model was:

Yijkl = µ + τi + sj + dk + εijkl

where Yijkl is the observation l in group i, sex j and dam k, µ is the overall mean, τi the fixed effect of group i (fat or lean), sj is the fixed effect of the sex j (male, or female), d is the random effect of the dam, εij the random error. Tukey’s multiple comparison test was used to determine which means amongst a set of means differ from the rest. Preliminary analyses showed no interaction between the group and sex for any traits; therefore, this term was not included in the model.

For different feed intake and feed conversion measurements at different periods, the model was:

Yij = µ + τi + εij

where all effects were the same as previously defined.

## 3. Results

The growth performances of rabbits divergently selected for total body fat content are shown in Table 2 and Table 3. The results highlighted that there was no significant difference in BW and DWG between the rabbits of the two divergently selected lines.

With regard to the FI, it was always numerically higher in the fat line, however, a statistical difference was observed only at weeks 9–10 (171 vs. 160 g/d, respectively; *p* < 0.05). Nevertheless, when considering the whole growing period, these differences did not highlight a higher FI in the fat line (5–11 w). However, the observed differences in FI between the lean and fat lines were sufficient to negatively affect the FCR at 7–8 w (3.95 vs. 3.58 for the fat and lean line, respectively; *p* < 0.001), at 10–11 w (5.58 vs. 4.98 for fat and lean line, respectively; *p* < 0.05) and in the whole growing period (3.86 vs. 3.61 for the fat and lean line, respectively; *p* < 0.001) (Table 3).

The effect of the divergent selection on rabbit carcass traits is depicted in Table 4. The divergent selection towards a leaner carcass (lean line) increased the proportion of the fore (*p* < 0.05) and the hind (*p* < 0.01) parts. However, the most successful effect of the divergent selection, throughout the in vivo total body fat content estimation by CT, was observed on the incidence of fat deposits to the reference carcass. In fact, the fat line exhibited carcasses with the highest proportion of both perirenal and scapular fat (*p* < 0.001), thus leading to the highest dissectible fat proportion (3.10 vs. 1.85% RC, for the fat and lean lines, respectively; *p* < 0.001).

**Figure 1:**
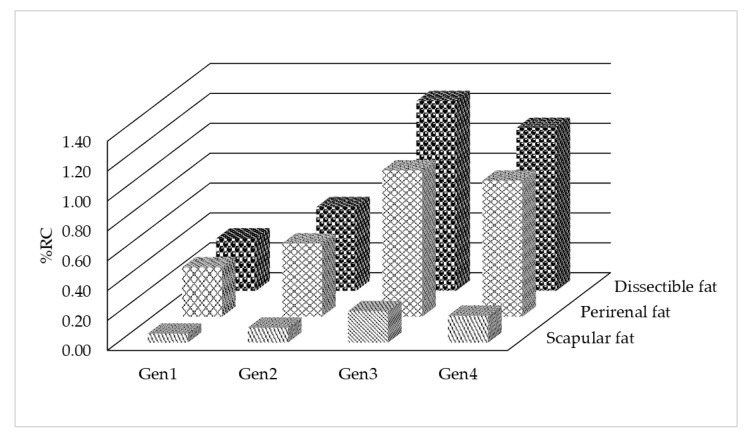
Differences in the fat depots (% of the reference carcass—%reference carcasses (RC)) between the fat and lean lines depending on the generation (Generations 1–4).

As shown in Figure 1, the differences in fat depots between the fat and lean lines increased from generation 1 to 4, with a higher rate of change until generation 3. The differences between lines were significant in each generation.

## 4. Discussion

As a result of divergent selection for the total body fat content, the perirenal and scapular fat depots were the highest in the fat line. This result confirms previous findings in mice [11,27] and in broiler chickens [8]. However, the magnitude of the fat content change varied according to the variable object of the divergent selection: rabbits divergently selected for intramuscular fat (IMF) content resulted in a symmetrical change of their IMF [28], whereas, as in the case of our study, rabbits were divergently selected for the total body fat content, and they exhibited an asymmetrical dissectible fat change (personal communication). The development of fat depots with the change of generation was not stable because of the effect of season. When the rabbits were slaughtered in hot season (summer) the feed intake and fat deposition decreased compared to other seasons.

After four generations of divergent selection, the ratio of perirenal fat, scapular fat and dissectible fat expressed to the RC was 1.74, 1.57 and 1.69 times higher in the fat line than in the lean one, respectively. Our results confirm, once more, that the divergent selection for fat content is an effective strategy to modulate carcass fatness, regardless of the animal species considered [5,6,7,8,11,27], and that the difference between lean and fat lines mainly depends on the number of generations considered. Bünger et al. [12] carried out a long-term divergent selection for body fatness in mice and after 60 generations, they obtained 22% and 4% body fat content in the fat and lean lines, respectively. These results also highlight that it is almost impossible to reduce the fat content over a specific threshold, but a further increase is feasible. This is one of the reasons why, even in longer divergent selection schemes, the fat tissue change is asymmetric in the two extreme lines. The asymmetrical response to selection is considered to be a possible limitation of divergent selection experiments; however, in this case, the fact that the selection was more effective in increasing the fat content of rabbit carcasses than decreasing it, should not be considered negatively. Rabbit meat is already considered a lean meat [29], thus even a little modification in the fat content can be considered a positive result in the consumer’s perspective.

In the study by Martínez-Álvaro et al. [9], it was highlighted that a divergent selection for the IMF of the Longissimus thoracis et lumborum muscle of rabbits, generated a positively correlated response also in the IMF of the Biceps femoris, Supraspinatus and Semimembranosus proprius muscles. When Zomeño et. al. [28,30] divergently selected rabbits for their IMF content, in parallel the perirenal fat content also changed, being 1.06 g higher in the high IMF than in the low IMF line. Thus, it appears that if one of the adipose depots is modified by divergent selection, a change also occurs in other depots in the same direction. In our experiment, the rabbits were selected for total body fat content, thus including all fat depots and according to our preliminary results, some meat cuts already showed differences in the lipids content already at generation 2 [24].

In rabbits, the divergent selection for meatiness also had a repercussion on body fatness. In fact, as it was already mentioned, a divergent selection for thigh muscles volume leads to significant differences in the perirenal fat (2.4% and 1.9% RC, *p* < 0.01) and scapular fat percentages (1.07% and 0.49% RC, *p* < 0.001), with lower values found in rabbits selected for higher thigh muscle volume [10].

In the current study, the lean line showed a higher proportion of the hind part of the carcass compared to the fat line, confirming that, when selecting for low fat depots, meatiness can increase. Despite this, it must also be mentioned that the loin proportion in the carcass was not affected by the divergent selection process.

Since the energy requirement for fat deposition is higher than that for protein (muscle) anabolism [31,32], the observed improvement of the FCR (5–11 weeks) observed in the lean line was expected, and confirmed previous results published by Baéza and Le Bihan-Duval [7]. Indirectly, Szendrő et al. also [10] observed a better FCR in rabbits divergently selected for higher muscle volume, due to their lower concurrent fat depots. Analogously to what was observed by Martinez et al. [11] and Baéza and Le Bihan-Duval [7], it seems that the divergent selection for body fat content has little or no effect on rabbit BW and DWG. The difference in feed intake appears to be primarily or exclusively due to the amount of fat deposits, so neither feed consumption nor the amount of fat deposits affected growth. As fat deposition is one of the most inherited traits [4,33], a minor change or invariance in the correlated traits such as growth rate is expected [10], because fat is a tissue of late deposition [4], while the growth of meat (body weight) is higher at a younger age [34,35]. However, when the rabbits were selected for ADG, a higher dissectible fat percentage of the carcass was found [36,37]. When animals are selected for weight gain, that would mainly exploit the genetic variance in appetite [38] and their feed intake increases [39]. In the case of selection for body fat content, appetite does not change, i.e., the feed intake may remain unchanged.

One of the purposes of the present study was to increase the total body fat content in a maternal line, in order to provide rabbit does with a high energy stores, useful for supporting their intensive reproductive life. In a parallel study of Kasza et al. [40] it was observed that, by the third generation of divergent selection, rabbit does of the fat group already had a higher number of total kits born (*p* < 0.1) and kits born alive (*p* < 0.01) than the rabbit does of the lean group. Furthermore, fat does had less stillborn kits (*p* < 0.05) and a higher litter size at 21 days of age compared to the lean ones. These preliminary findings suggest that the divergent selection programme could be promising in this sense.

## 5. Conclusions

The divergent selection for total body fat content in live rabbits, based on CT measurement, was effective in decreasing (lean line) and increasing (fat line) the amount of fat depots. Selection for lower total body fat content could be useful for terminal male lines, thus generating meat-producing rabbits characterized by a lower FCR and less slaughter waste. On the contrary, the selection for higher total body fat content could be an advantage for rabbit does in providing energy reserves that they can mobilize when needed, i.e., at the end of pregnancy or close to the lactation peak. Nevertheless, the efficacy of the fat line in the reproductive ability of the rabbit does needs to be confirmed by further research.

## Figures and Tables

**Table 1:** Scheme of the divergent selection process.

Generation	Line	Female				Male			
		CT Scanned		Selected		CT Scanned		Selected	
		*n*	Fat Index	*n*	Fat Index	*n*	Fat Index	*n*	Fat Index
1	Lean	209	75.2 ± 20.0	72	54.4 ± 9.2	142	68.0 ± 18.2	35	47.0 ± 9.9
	Fat			72	96.4 ± 11.7			34	89.7 ± 10.7
2	Lean	96	68.8 ± 19.3	67	59.2 ± 10.9	80	69.2 ± 18.8	40	57.0 ± 13.2
	Fat	105	75.6 ± 17.6	64	86.0 ± 12.0	80	78.5 ± 18.8	40	90.4 ± 14.9
3	Lean	180	61.5 ± 15.5	61	49.1 ± 10.5	149	65.8 ± 14.8	47	56.6 ± 8.97
	Fat	187	75.5 ± 16.7	61	90.6 ± 10.8	142	78.5 ± 17.3	47	86.6 ± 13.3
4	Lean	105	77.4 ± 16.3	55	67.5 ± 11.7	112	75.0 ± 15.9	44	63.7 ± 10.7
	Fat	104	101 ± 18.5	57	111 ± 15.1	108	94.5 ± 19.9	42	109 ± 15.6

**Table 2:** Least square means (±SE) of body weight and daily weight gain of lean and fat growing rabbits.

	Lean	Fat	*p*-Value
N of rabbits	60	60	
Body weight, g			
week 5	892 ± 15.5	873 ± 14.4	0.348
week 6	1288 ± 19.4	1252 ± 18.4	0.177
week 7	1624 ± 23.3	1593 ± 21.9	0.329
week 8	1920 ± 27.2	1874 ± 25.8	0.223
week 9	2164 ± 29.6	2125 ± 28.5	0.344
week 10	2402 ± 33.5	2371 ± 32.3	0.493
week 11	2607 ± 37.4	2564 ± 35.8	0.403
Daily weight gain, g/day			
week 5–6	49.5 ± 1.00	47.5 ± 0.96	0.140
week 6–7	47.8 ± 1.08	48.7 ± 1.00	0.544
week 7–8	42.8 ± 1.12	40.3 ± 1.09	0.193
week 8–9	35.0 ± 1.13	35.9 ± 1.12	0.549
week 9–10	33.9 ± 1.25	35.1 ± 1.24	0.492
week 10–11	34.6 ± 1.32	32.4 ± 1.31	0.255
weeks 5–11	40.9 ± 0.71	40.3 ± 0.69	0.557

**Table 3:** Least square means (±SE) of feed intake and feed conversion ratio of the lean and fat growing rabbits.

	Lean	Fat	*p*-Value
N of cages	20	20	
Feed intake, g/day			
week 5–6	129 ± 3.09	131 ± 3.09	0.677
week 6–7	131 ± 3.77	137 ± 3.77	0.257
week 7–8	152 ± 2.44	158 ± 2.44	0.075
week 8–9	159 ± 2.51	164 ± 2.51	0.175
week 9–10	160 ± 3.49	171 ± 3.49	0.032
week 10–11	167 ± 3.63	170 ± 3.63	0.564
weeks 5–11	150 ± 2.21	155 ± 2.21	0.085
Feed conversion ratio			
week 5–6	2.59 ± 0.09	2.71 ± 0.09	0.318
week 6–7	2.63 ± 0.07	2.79 ± 0.07	0.084
week 7–8	3.58 ± 0.07	3.95 ± 0.07	<0.001
week 8–9	4.52 ± 0.10	4.70 ± 0.10	0.179
week 9–10	4.72 ± 0.14	5.02 ± 0.14	0.140
week 10–11	4.98 ± 0.17	5.58 ± 0.17	0.015
weeks 5–11	3.61 ± 0.03	3.86 ± 0.03	<0.001

**Table 4:** Least square means (±SE) of the carcass traits of lean and fat growing rabbits.

	Lean	Fat	*p*-Value
N of rabbits	60	60	
Slaughter weight, g	2606 ± 39.2	2527 ± 37.0	0.144
Hot carcass, g	1573 ± 23.1	1531 ± 21.9	0.200
Chilled carcass, g	1534 ± 22.7	1495 ± 21.6	0.219
Reference carcass, g	1291 ± 19.0	1262 ± 18.0	0.266
Dressing out percentage:			
Hot carcass	60.4 ± 0.25	60.6 ± 0.24	0.489
Chilled carcass	58.9 ± 0.25	59.1 ± 0.24	0.392
Reference carcass	49.6 ± 0.26	49.9 ± 0.26	0.355
Percentage to the chilled carcass:			
Reference carcass	84.2 ± 0.15	84.4 ± 0.14	0.435
Head	8.79 ± 0.12	8.27 ± 0.11	0.002
Thoracic cage organs	1.56 ± 0.04	1.65 ± 0.04	0.125
Liver	4.42 ± 0.11	4.65 ± 0.11	0.151
Kidneys	1.04 ± 0.2	1.08 ± 0.02	0.185
Percentage to the reference carcass:			
Fore part	30.8 ± 0.18	30.1 ± 0.18	0.020
Mid part	30.3 ± 0.18	30.5 ± 0.18	0.424
Hind part	36.9 ± 0.20	36.2 ± 0.19	0.006
Perirenal fat	1.39 ± 0.10	2.37 ± 0.10	<0.001
Scapular fat	0.49 ± 0.04	0.75 ± 0.04	<0.001
Dissectible fat	1.85 ± 0.13	3.10 ± 0.13	<0.001
